# Long-Term Outcomes of Strabismus Surgery in Children with Cerebral Palsy: Focus on Consecutive Exotropia

**DOI:** 10.3390/children13080974

**Published:** 2026-07-23

**Authors:** Ceren Gürez, Zahid Hüseyinhan

**Affiliations:** Department of Ophthalmology Clinic, Beyoglu Eye Training and Research Hospital, Health of Sciences University, 34437 Istanbul, Turkey; han1969@live.com

**Keywords:** cerebral palsy, esotropia, exotropia, consecutive exotropia, strabismus surgery, Botulinum toxin A

## Abstract

**Highlights:**

**What are the main findings?**

- Long-term ocular alignment was successfully achieved in approximately two-thirds of children with cerebral palsy following individualized interventional treatment.
- Consecutive exotropia occurred exclusively after treatment of esotropia, whereas residual deviation was significantly more common after exotropia treatment.

**What are the implications of the main findings?**

- Children with cerebral palsy require long-term postoperative follow-up because ocular alignment may change substantially over time.
- Individualized treatment strategies, including strabismus surgery with or without Botulinum toxin A, can provide satisfactory long-term outcomes, although postoperative alignment instability remains an important clinical challenge.

**Abstract:**

Background/Objectives: To evaluate the long-term outcomes of strabismus treatment in children with cerebral palsy (CP) and to investigate factors associated with postoperative alignment success and the development of consecutive strabismus. Methods: The retrospective, single-center study included 73 children with CP and strabismus who were followed from January 2015 to December 2024. Demographic characteristics, ophthalmological findings, treatment methods, and postoperative outcomes were assessed. Ocular deviation was measured through prism and alternate cover testing or a modified Krimsky test, and surgical success was defined as a final deviation of ≤10 prism diopters (PD). Patients were assessed at the patient level. Comparisons between exotropic and esotropic patients and between patients who had consecutive exotropia and those who had successful alignment were made using appropriate statistical tests. Results: Study participants included 55 patients with esotropia (75.3%) and 18 patients with exotropia (24.7%). The mean follow-up period was 32.6 ± 7.18 months. A successful ocular alignment was achieved in 32 of 46 patients (69.6%) with esotropia and in 10 of 17 patients (58.8%) with exotropia following surgical treatment and/or BTA. Consecutive exotropia developed exclusively in patients initially treated for esotropia (39.1% vs. 0%, *p* = 0.001). Patients with consecutive exotropia underwent surgery at a younger age and had larger preoperative deviation angles than those who had successful alignment, but these differences were not statistically significant. Conclusions: Strabismus treatment provides satisfactory long-term motor alignment in many children with cerebral palsy. However, consecutive exotropia following esotropia surgery remains a common postoperative condition and is the principal source of alignment instability in this population. Younger surgical age and larger preoperative deviations were more common among patients with consecutive exotropia, but no statistically significant associations between the evaluated clinical variables and consecutive exotropia were identified in the exploratory univariate analyses.

## 1. Introduction

Cerebral palsy (CP) is a neurodevelopmental disorder characterized by abnormal muscle tone, movement problems, and deficits in motor coordination [1]. The global prevalence of CP is 2.1 per 1000 live births; however, in Turkey, it is 4.4 per 1000 live births [2,3]. CP is typically classified into spastic, ataxic, athetoid, and atonic subtypes, with the spastic type being the most common. This type is also associated with ocular problems. Comorbid conditions include epilepsy, intellectual disability, visual and auditory impairments, urinary and fecal incontinence, spinal and hip disorders, and communication challenges [1,3]. Between 50% and 90% of individuals with CP have ophthalmological problems, such as severe refractive errors, strabismus, amblyopia, nystagmus, and cortical visual impairment [4,5]. In particular, strabismus is more common because it affects the neuromuscular control of the extraocular muscles.

Visual impairment is one of the most common associated conditions in children with CP. Reported ophthalmic manifestations include refractive errors, amblyopia, nystagmus, cortical visual impairment, optic nerve abnormalities, and strabismus. Recent systematic reviews have confirmed that strabismus is among the most frequent ocular disorders in this population, with esotropia occurring more commonly than exotropia, particularly in children with spastic CP [2]. Abnormal supranuclear control of eye movements, impaired binocular sensory development, defective vergence mechanisms, and reduced fusional capacity contribute to the high prevalence of ocular misalignment and its persistence throughout childhood.

Management of strabismus in children with CP remains challenging. Preoperative evaluation is often complicated by limited cooperation, developmental delay, impaired communication, variable muscle tone, and reduced binocular sensory function. Consequently, the surgical response is less predictable than in neurologically typical children, and postoperative alignment instability is more common. Conventional strabismus surgery remains the mainstay of treatment, whereas botulinum toxin A (BTA) has been used either as a primary treatment for selected patients or as an adjunct to surgery to reduce surgical dosage and improve postoperative alignment [6,7,8].

Several studies have reported acceptable short-term motor outcomes following strabismus surgery in children with CP [7,8,9]. However, maintaining long-term ocular alignment remains difficult because postoperative exotropic drift, residual deviation, and consecutive exotropia occur more frequently than in neurologically normal children. Despite the high prevalence of strabismus in children with CP, relatively few studies have evaluated long-term outcomes of different treatment approaches in routine clinical practice. In particular, data regarding the occurrence of residual deviation, consecutive strabismus, reoperation, and long-term motor alignment following both surgical and nonsurgical management remain limited.

Therefore, the present study aimed to evaluate the long-term motor outcomes of surgical and nonsurgical treatment of strabismus in children with cerebral palsy and to investigate clinical factors associated with postoperative ocular alignment, residual deviation, and the development of consecutive strabismus.

## 2. Methods

### 2.1. Study Design

This retrospective single-center observational study was conducted at the Strabismus Unit of the Department of Ophthalmology of our institution. The study protocol was approved by the Clinical Research Ethics Committee of Health Sciences University, Prof. Dr. Cemil Taşçıoğlu City Hospital (Protocol No. 20/05.02.2024). The Declaration of Helsinki was followed, and written informed consent was obtained from the parents or legal guardians of all participants.

### 2.2. Patient Selection

A total of 73 children diagnosed with cerebral palsy and strabismus who were evaluated between January 2015 and January 2024 were included. Cerebral palsy had been diagnosed by pediatric neurologists according to established clinical criteria before ophthalmologic evaluation. Patients with previous ocular surgery, ocular trauma, incomplete medical records, insufficient follow-up, absence of a confirmed diagnosis of cerebral palsy, or without manifest strabismus were excluded.

### 2.3. Ophthalmological Examination

All patients underwent a comprehensive ophthalmological examination including best-corrected visual acuity (BCVA), cycloplegic refraction, anterior and posterior segment evaluation, ocular motility assessment, and measurement of ocular deviation. BCVA was assessed using a Snellen visual acuity chart at 5 m whenever possible and converted to logarithm of the minimum angle of resolution (logMAR) values for statistical analysis. In children with limited cooperation, visual function was evaluated using fixation behavior and object-tracking ability.

Ocular deviation was measured using the prism and alternate cover test at both distance and near fixation. In patients with poor cooperation, the modified Krimsky test was performed. To reduce measurement variability related to limited cooperation, ocular alignment was assessed over three to five separate visits before treatment, and the most consistent measurements were used for analysis.

### 2.4. Unit of Analysis

Because treatment decisions and postoperative outcomes were determined for each individual child, the patient rather than the eye was considered the unit of analysis. Surgical success, residual deviation, and consecutive strabismus were therefore evaluated on a per-patient basis.

### 2.5. Outcome Measures

Postoperative ocular alignment was the primary outcome measure. Secondary outcome measures included the initial surgical success rate, final motor alignment after any additional intervention, residual deviation, reoperation, and the occurrence of consecutive strabismus.

Initial surgical success was defined as ocular alignment within 10 prism diopters (PD) following the first intervention. Final success was defined as ocular alignment within 10 PD at the last follow-up visit after completion of all planned treatments, including additional surgery or botulinum toxin injection when required.

### 2.6. Statistical Analysis

Statistical analyses were performed using SPSS software (version 20.0; SPSS Inc., Chicago, IL, USA). Continuous variables were presented as mean ± standard deviation or median (interquartile range) according to data distribution, whereas categorical variables were expressed as frequencies and percentages. Normality was assessed using the Shapiro–Wilk test.

Comparisons between patients with esotropia and exotropia were performed using the independent-samples *t* test or the Mann–Whitney *U* test for continuous variables and the chi-square or Fisher’s exact test for categorical variables. Preoperative and postoperative deviation angles were compared using paired-samples tests, as appropriate. A two-sided *p* value < 0.05 was considered statistically significant.

Due to the relatively small sample size, particularly the limited number of consecutive exotropia patients, multivariate regression analysis was not considered statistically appropriate due to the increased risk of overfitting. In order to determine whether clinical characteristics may be associated with postoperative outcomes, exploratory univariate analyses were undertaken.

## 3. Results

There were 73 children (34 females and 39 males) with cerebral palsy and strabismus with a mean age of 6.28 ± 2.73 years (2–12 years) in the study. The mean age at intervention was 3.69 ± 2.12 years, and the mean follow-up duration was 32.6 ± 7.18 months. Baseline demographic characteristics are given in Table 1.

Esotropia was the predominant type of strabismus and was present in 55 patients (75.3%), whereas 18 patients (24.7%) had exotropia. The mean spherical equivalent refractive error was +2.27 ± 1.86 D in the right eye and +2.19 ± 1.77 D in the left eye. Mean best-corrected visual acuity was 0.73 ± 0.68 logMAR in the right eye and 0.69 ± 0.53 logMAR in the left eye.

Patients with esotropia underwent active interventional treatment (strabismus surgery and BTA, or BTA alone), whereas nine patients with small-angle deviations were treated conservatively. In the exotropia group, 17 patients underwent surgical treatment and one patient was managed conservatively. The distribution of treatment modalities is summarized in Table 2.

Patients with exotropia presented at an older age than those with esotropia (8.63 ± 2.19 vs. 5.87 ± 2.62 years, *p* = 0.006). Likewise, the mean age at intervention was significantly greater in the exotropia group (5.50 ± 3.21 vs. 3.39 ± 1.78 years, *p* = 0.007). The duration of follow-up also differed significantly between the two groups (*p* = 0.022). The mean preoperative near deviation was 30.38 ± 10.09 prism diopters (PD) in the esotropia group and 35.63 ± 5.27 PD in the exotropia group. Neither the preoperative nor the final postoperative deviation differed significantly between groups (*p* = 0.236 and *p* = 0.161, respectively) (Table 3).

A successful ocular alignment (≤10 PD at the final follow-up) was achieved in 32 of 46 patients (69.6%) with esotropia and in 10 of 17 patients (58.8%) with exotropia following surgical treatment and/or BTA. Residual deviation was significantly more frequent in the exotropia group (47.1% vs. 10.9%, *p* = 0.004). Consecutive exotropia developed exclusively in patients initially treated for esotropia (39.1% vs. 0%, *p* = 0.001). There was no significant difference between the groups when it came to the number of patients requiring reoperations (15.2% for esotropia and 17.6% for 34 exotropia) (*p* = 1.000). The detailed postoperative outcomes are presented in Table 4.

To investigate factors associated with consecutive exotropia, patients who developed consecutive exotropia were compared with those who achieved successful ocular alignment. Although patients with consecutive exotropia tended to undergo intervention at a younger age and had larger preoperative deviation angles, none of the evaluated clinical variables showed a statistically significant association in the exploratory univariate analyses (Table 5). Similarly, botulinum toxin A use was not significantly associated with the development of consecutive exotropia (33.3% vs. 40.6%, Fisher’s exact test, *p* = 0.78). Because of the limited number of patients with consecutive exotropia, multivariable regression analysis was not performed.

Among the nine patients with esotropia managed conservatively, spontaneous improvement in ocular alignment was observed during follow-up, and none subsequently required surgical intervention.

## 4. Discussion

Strabismus is one of the most common ophthalmic manifestations of cerebral palsy, reflecting abnormalities in supranuclear ocular motor control, impaired binocular sensory development, and reduced fusional capacity. Consequently, postoperative alignment in these children is generally less predictable than in neurologically typical patients. Recent reviews have emphasized that both sensory deficits and abnormal motor control contribute to the increased variability of surgical outcomes in this population. Nearly half of children with cerebral palsy develop strabismus, with esotropia being the predominant subtype in most populations. Persistent abnormalities in ocular motor control and binocular vision likely contribute to the increased variability of postoperative alignment in these patients [6,7,8,9,10,11,12].

The present study evaluated the long-term motor outcomes of strabismus treatment in children with cerebral palsy and demonstrated that satisfactory ocular alignment could be achieved in a considerable proportion of patients following active intervention. However, postoperative alignment instability remained an important clinical challenge, particularly among patients with esotropia. Consecutive exotropia occurred exclusively after treatment of esotropia, whereas residual deviation was significantly more frequent in patients with exotropia. Although younger age at intervention and larger preoperative deviation were more frequently observed among patients who developed consecutive exotropia, none of the evaluated clinical variables was significantly associated with consecutive exotropia in the exploratory univariate analyses.

Previous studies have reported surgical success rates ranging from approximately 45% to 70% in children with cerebral palsy, depending on the type of strabismus, surgical technique, duration of follow-up, and definition of success [8,13,14]. In our cohort, successful motor alignment at the final follow-up was achieved in 69.6% of patients with esotropia and 58.8% of those with exotropia following active interventional treatment. These findings are comparable with previously published series and support the effectiveness of strabismus surgery in this challenging patient population despite the presence of neurological impairment.

A recent systematic review likewise emphasized that although surgical correction generally provides satisfactory ocular alignment, long-term stability remains less predictable in children with cerebral palsy than in neurologically typical children [2].

One of the most clinically relevant findings of our study was the high frequency of consecutive exotropia after correction of esotropia. Similar observations have been reported previously by Ma et al. and Leite et al., who demonstrated that postoperative exotropic drift represents one of the principal causes of long-term alignment instability in children with cerebral palsy [7,8,9]. Several mechanisms have been proposed to explain this phenomenon, including exaggerated responses to medial rectus recession, impaired vergence adaptation, reduced sensory fusion, and progressive changes in muscle tone associated with cerebral palsy. Collectively, these factors may predispose children with cerebral palsy to postoperative overcorrection even when conventional surgical dosages are used [15].

Residual deviation remained significantly more common after exotropia surgery than after esotropia correction. Although the overall alignment success was comparable between the two groups, this finding suggests that achieving stable correction of exotropia in children with cerebral palsy remains challenging. Similar observations have been reported in previous studies, indicating that postoperative alignment variability is not limited to esotropia but may also affect exotropia because of the underlying neurological disorder [4,13].

Botulinum toxin A has become an increasingly accepted component of multidisciplinary management in children with cerebral palsy. Although most evidence concerns treatment of spasticity, recent reviews also support its favorable safety profile and individualized use in pediatric patients, including ophthalmic indications when appropriate [8,15].

Botulinum toxin A (BTX) has been increasingly used as either a primary treatment or an adjunct to conventional strabismus surgery in selected children with cerebral palsy. Tugcu et al. reported that BTA achieved satisfactory motor alignment in children with neurological impairment, particularly in those with esotropia, supporting its role as a valuable treatment option in carefully selected patients [8]. In the present study, the decision to use BTA was individualized according to the magnitude of deviation, clinical characteristics, and surgeon preference rather than a standardized treatment protocol. Consequently, meaningful comparisons between specific treatment modalities were not feasible because of the limited number of patients in each procedural subgroup. Consistent with recent reviews emphasizing that the effectiveness of BTA depends largely on patient selection, deviation magnitude, and injection technique rather than a uniform treatment algorithm [16,17], we found no statistically significant association between BTA use and the development of consecutive exotropia in the exploratory univariate analysis. Because of the relatively small sample size and the individualized treatment strategy, it is important to interpret this finding with caution.

In the exploratory univariate analysis, BTX use was not significantly associated with the development of consecutive exotropia. However, this finding should be interpreted cautiously because treatment allocation was individualized rather than randomized, and the sample size was insufficient to evaluate the independent effect of BTX.

The strengths of this study include one of the larger single-center cohorts of children with cerebral palsy undergoing long-term follow-up for strabismus treatment, inclusion of both surgical and botulinum toxin interventions, and evaluation of clinically relevant outcomes including residual deviation, consecutive strabismus, and reoperation. These findings provide practical information regarding long-term alignment stability in routine clinical practice.

There are, however, a few limitations to be noted. As a first concern, there is a possibility of selection and information bias due to the retrospective design. Second, the number of patients within individual treatment subgroups was insufficient to permit reliable comparisons between different surgical procedures or to perform multivariable regression analyses. Third, cerebral palsy subtype, severity, and functional classification were not consistently available and therefore could not be incorporated into outcome analyses. Finally, this was a single-center study, which may limit the generalizability of the findings. Prospective multicenter studies with standardized treatment protocols and larger patient cohorts are required to better define prognostic factors for long-term ocular alignment in children with cerebral palsy.

## 5. Conclusions

In conclusion, active interventional treatment achieved satisfactory long-term ocular alignment in many children with cerebral palsy. However, postoperative alignment instability remains common, particularly consecutive exotropia following esotropia correction and residual deviation after exotropia surgery. Although none of the evaluated clinical variables was significantly associated with consecutive exotropia in the exploratory univariate analyses, careful long-term follow-up remains essential because ocular alignment may change substantially over time in this complex patient population.

## Figures and Tables

**Table 1:** Demographic and Clinical Characteristics of the Patients.

Parameter	Value
Sex (Female/Male)	34/39
Mean Age (years)	6.28 ± 2.73 (range: 2–12)
Mean Age at Surgery (years)	3.69 ± 2.12 (range: 1–9)
Mean Follow-up Period (months)	32.6 ± 7.18 (range: 12–48)
Type of Misalignment	Esotropia/Exotropia: 55/18
Refractive Error (Spherical, D)	OD: +2.27 ± 1.86 (–5.50 to +5.75) OS: +2.19 ± 1.77 (–4.50 to +5.25)
Best-Corrected Visual Acuity (logMAR)	OD: 0.73 ± 0.68 OS: 0.69 ± 0.53

**Table 2:** Treatment Modalities According to Strabismus Type.

Strabismus Type	BMR	BMR + BTX	BTX	MRR–LRR	BMR + Faden	LRR–MRR	BLR	No Surgery
Esotropia (ET)	23	10	9	3	3	0	0	7
Exotropia (XT)	0	0		0	0	7	10	1

Abbreviations: BMR = Bilateral Medial Rectus muscle recession, BMR + BTX = Bilateral Medial Rectus recession with Botulinum Toxin A injection, BTX: Bilateral Medial Rectus muscle Botulinum Toxin A injection, MRR–LRR = Unilateral Medial Rectus recession and Lateral Rectus resection, BMR + Faden = Bilateral Medial Rectus recession with Faden operation, LRR–MRR = Unilateral Lateral Rectus recession and Medial Rectus resection, BLR = Bilateral Lateral Rectus recession, No Surgery = Patients managed conservatively.

**Table 3:** Clinical Characteristics of Patients with Esotropia and Exotropia.

Variable	ET (n = 55)	XT (n = 18)	*p* Value
Age (years)	5.87 ± 2.62	8.63 ± 2.19	0.006
Age at intervention (years)	3.39 ± 1.78	5.50 ± 3.21	0.007
Follow-up duration (months)	34.6 ± 6.82	31.3 ± 4.17	0.022
Preoperative deviation (PD)	30.38 ± 10.09	35.63 ± 5.27	0.236
Final deviation (PD)	11.80 ± 8.88	13.25 ± 10.36	0.161
Initial success (%)	58.2	55.6	0.680
Final success (%)	69.1	61.1	0.173

**Table 4:** Postoperative motor outcomes of patients undergoing active interventional treatment for esotropia and exotropia.

Outcome	ET (n = 46)	XT (n = 17)	*p* Value
Successful alignment (≤10 PD)	32 (69.6%)	10 (58.8%)	0.549
Residual deviation	5 (10.9%)	8 (47.1%)	0.004
Consecutive deviation	18 (39.1%)	0 (0%)	0.001
Reoperation required	7 (15.2%)	3 (17.6%)	1.000

**Table 5:** Comparison of Esotropic Patients With Consecutive Exotropia and Successful Alignment.

Variable	Consecutive Exotropia (n = 18)	Successful Alignment (n = 32)	Statistical Test	*p* Value
Age at intervention (years)	3.1 ± 1.2	3.8 ± 1.6	Mann–Whitney U	0.21
Follow-up duration (months)	38.41 ± 5.35	34.51 ± 3.76	Mann–Whitney U	0.54
Preoperative near deviation (PD)	31.42 ± 6.27	28.48 ± 5.18	Mann–Whitney U	0.18
Botulinum toxin A use, n (%)	6 (33.3%)	13 (40.6%)	Fisher’s exact	0.78

Continuous variables were compared using the independent-samples *t* test or Mann–Whitney U test according to data distribution. Categorical variables were compared using Fisher’s exact test.

## Data Availability

The data presented in this study are available on request from the corresponding author due to privacy.
